# Ternary Quantum Dots in Chemical Analysis. Synthesis and Detection Mechanisms

**DOI:** 10.3390/molecules26092764

**Published:** 2021-05-08

**Authors:** Raybel Muñoz, Eva M. Santos, Carlos A. Galan-Vidal, Jose M. Miranda, Aroa Lopez-Santamarina, Jose A. Rodriguez

**Affiliations:** 1 Area Academica de Quimica, Universidad Autonoma del Estado de Hidalgo, Carr. Pachuca-Tulancingo Km. 4.5, Mineral de la Reforma, Hidalgo 42184, Mexico; raybel_munoz@uaeh.edu.mx (R.M.); emsantos@uaeh.edu.mx (E.M.S.); galanv@uaeh.edu.mx (C.A.G.-V.); 2 Laboratorio de Higiene Inspección y Control de Alimentos, Dpto. de Quimica Analitica, Nutricion y Bromatologia, Facultad de Veterinaria, Pabellon, 4 p.b. Campus Universitario, Universidad de Santiago de Compostela, 27002 Lugo, Spain; josemanuel.miranda@usc.es (J.M.M.); aroa.lopez.santamarina@usc.es (A.L.-S.)

**Keywords:** ternary QDs, FRET, PET, enhancement, analytical methodologies

## Abstract

Ternary quantum dots (QDs) are novel nanomaterials that can be used in chemical analysis due their unique physicochemical and spectroscopic properties. These properties are size-dependent and can be adjusted in the synthetic protocol modifying the reaction medium, time, source of heat, and the ligand used for stabilization. In the last decade, several spectroscopic methods have been developed for the analysis of organic and inorganic analytes in biological, drug, environmental, and food samples, in which different sensing schemes have been applied using ternary quantum dots. This review addresses the different synthetic approaches of ternary quantum dots, the sensing mechanisms involved in the analyte detection, and the predominant areas in which these nanomaterials are used.

## 1. Introduction

Colloidal nanocrystals of semiconductor materials, or most commonly referred to as quantum dots (QDs), are inorganic fluorescent nanoparticles with sizes in the range of 1 to 20 nm (dimensions smaller than the exciton Bohr radius), generally coated with an organic ligand [1]. At these dimensions, QDs exhibit unique spectroscopic properties such as broad absorption and narrow emission spectra, high extinction coefficients, high quantum yields (QY), photostability, and large surface area, characteristics that are not observed in the bulk materials [2].

Quantum dots are tunable in their spectroscopical properties; therefore, it is possible to adjust their absorption and emission maximum wavelength in the visible and near infrared spectrum by modifying their size during the synthesis [3]. This characteristic along with their possible surface interactions with different compounds make them useful to develop chemical sensing strategies. In this sense, several analytical methodologies have been proposed for detection of a large number of analytes contained in different kind of matrices: drug, biological [4], food [5], environmental [6], etc.

Different QDs have been synthesized through the years, but the most used in chemical analysis methodologies are binary QDs. They are composed mainly of Cd (II) or Pb (II) and a chalcogenide such as S, Se, or Te. Unfortunately, since these metal ions contained in the nanoparticles exhibit high toxicity to humans and cause contamination to the environment, it is necessary to find alternative materials with similar advantages [7].

An alternative to Cd- and Pb-based quantum dots are I-III-VI Ternary Quantum Dots, which are semiconductors with a multicomponent structure composed of three different elements: a metal of the IB group (Cu or Ag), a metal of the IIIB (commonly In, Sn, Al, or Ga), a chalcogen of the VIA group (S, Se, or Te), and a ligand to stabilize the structure in aqueous media. These nanomaterials present similar optical properties with lower toxicity [8]. Nanomaterials such as CuInS_2_, AgInS_2_, or CuInSe_2_ have been recently studied and they showed the same size-dependent spectroscopic properties. Different synthesis protocols have been proposed to obtain the desired optical properties [9].

The use of ternary QDs in analytical applications has increased in the last decade. When a methodology is designed, different variables need to be considered as the spectroscopical properties’ characteristics and the interactions with the analyte [10]. Different protocols have been proposed for the synthesis of aqueous ternary quantum dots, in which the precursors or components react to form the nanoparticle with the desired size (therefore, the spectroscopic properties) and surface ligand. The synthetic reactions can be performed by hydrothermal, solvothermal, or microwave conditions. The synthesis protocol confers different physical and chemical properties [11].

Commonly, the analytical methodologies are based in spectroscopic changes of QDs due their interaction with an analyte; therefore, it is important to consider the analyte chemistry and the mechanisms of reaction with QDs. Some common changes are: quenching of the emission signal due to QDs’ changes on the surface, quenching due to an energy transfer from QDs to an acceptor referred to as Foster resonance energy transfer (FRET), and enhancing of the emission signal due to a passivation of surface traps, among others [10].

This review contains the state-of-the-art of ternary quantum dots application in chemical analysis, including the advantages and disadvantages of the different synthetic protocols, the interactions between analytes and QDs, their current application on chemical analysis, and the future trends of these novel materials.

## 2. Synthesis of Ternary Quantum Dots

**Figure 1:**
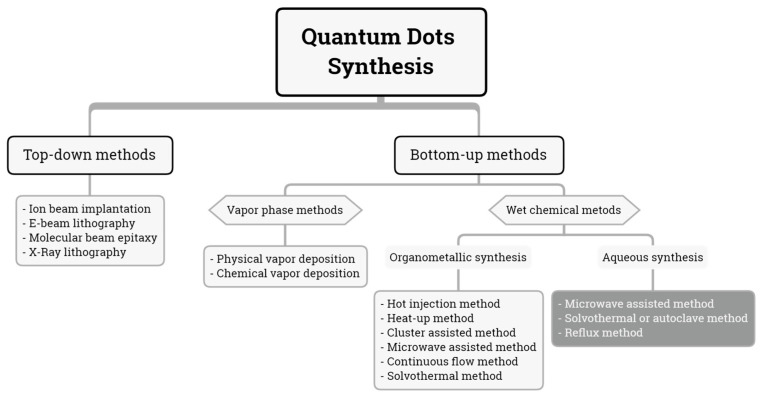
Methods for QD synthesis.

**Figure 2:**
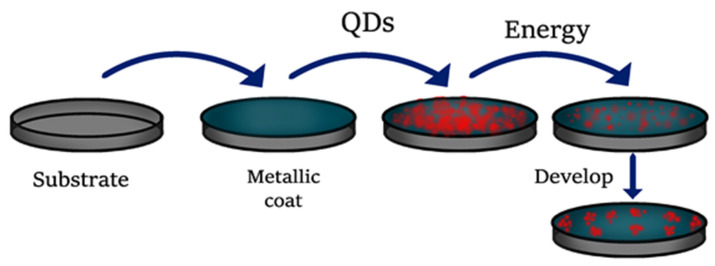
General procedure for synthesis of QDs by top-down techniques.

There are two approaches to obtain QDs (Figure 1), top-down and bottom-up methods. The former method involves strategies based on the size reduction of bulk materials under the influence of a beam [12] (Figure 2). Techniques such as ion beam implantation, electron beam lithography, molecular beam epitaxy, and x-ray lithography are included in this first category [13] and allow us to synthesize nanoparticles of InGaAs/GaAs [14], InGaN/GaN [15], among others. In a typical lithographic process, the QDs are deposited onto a silicon substrate coated with gold, to further draw a pattern by lithography over the QDs layer. The excess of QDs is removed using a solvent [16].

Different QDs with specific structures can be obtained applying these technologies, such as nanowires [17], circular [18], and pyramid-shaped nanoparticles [19] in a large range of sizes (3–50 nm but commonly of 30 nm). QDs synthesized by these methodologies are used mainly for photovoltaic applications, optoelectronics (LEDs), and energy storage in solar cells [16].

**Figure 3:**
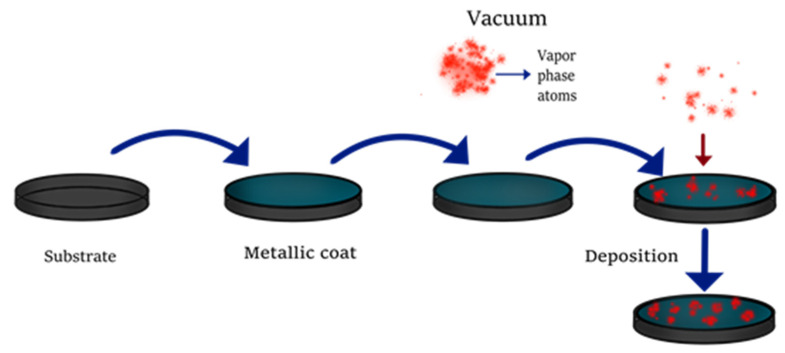
Synthesis of QDs by vapor phase methods.

The second approach is the button-up method [13], in which QDs are synthesized using chemical precursors. This category can be subdivided into vapor phase and wet chemical methods [20]. Vapor phase methods (VPM, Figure 3) generate precursors in vapor phase to further condense them atom by atom on a substrate and grow the nanoparticle [21]. Physical and chemical deposition are the most common strategies of VPM in which ternary QDs, such as InGaAs [22] and InGaN/GaN [23], and binary QDs, such as ZnSe/ZnS [24], InGaAs/InAs [25], InAs, CdSe, or CdTe [26], have been synthesized by these techniques and their main applications are in solar cells and optoelectronics [27].

In wet chemical methods, the synthesis of QDs occurs in a solvent, through the reaction of precursors at different temperatures. These precursors are either organometallic, which requires organic solvents for the synthesis (used in strategies such as the hot injection, heat-up cluster-assisted methods, microwave assisted synthesis, continuous flow method, and solvothermal method), or inorganic, which is mostly used for colloidal aqueous synthesis [11].

CdS, CdTe, CdSe, ZnS, ZnSe, InP, InAs, PbSe, PbS, PbTe, CdZnSe, AgInS_2_, CuInS_2_ are examples of QDs synthesized through organometallic synthesis. Compounds such as dimethylcadmium, diethylzinc, bis(tert-butyldimethylsilyl) telluride, trimethylsilyl, trioctylphosphine selenide, indium acetate, tris(trimethylsilyl)phosphine, bis(trimethylsilyl)selenide, lead acetate are common precursors to obtain QDs when these methodologies are employed [28]. The solvents commonly used are tri-n-octylphosphine oxide, tri-n-octylphosphine, hexadecylamine, dodecylamine [29].

The relevance of these methodologies remains due to the advantages that are presented such as the high %QY, the lower cost of equipment compared to the top-down methods, rapidness of the reactions, the lower size of QDs achieved (few nanometers vs. 30 nm in top-down methods), and that they can be employed in biomedical applications and analytical detections. Unfortunately, the disadvantages of these methodologies are the temperature requirements (up to 250 °C) and the presence of solvent residues [11].

Aqueous synthesis of ternary quantum dots is based on the reaction of inorganic ion precursors with chalcogenides and the stabilization of the resulting nanoparticle with a capping ligand. This process requires heating in the presence of inert gas in order to avoid the formation of metal oxides. This alternative has been attractive to designing chemical analysis methodologies due to the advantages that it presents, such as compatibility with sample matrices, lack of contaminant of organic solvents, milder conditions of synthesis, and in some cases, higher quantum yields (%QY) [30]. Considering the importance of QDs obtained by aqueous synthesis, the following section describes the most common synthetic protocols employed to obtain ternary QDs.

### 2.1. Aqueous Synthesis of Quantum Dots

The most common metallic precursors used for the synthesis of ternary QDs (AgInS_2_, CuInS_2_, and CuInSe_2_) are silver nitrate, cupric chloride, indium (III) nitrate, and indium (III) chloride as the metal source, and sodium sulfide or metallic selenide as the chalcogenide source, although it is common to use thiourea (CS(NH_2_)_2_), due the degradation at high temperatures into sulfide ion [31].

Different capping agents have been used in the synthesis of ternary quantum dots, but the thiol-containing compounds are used more frequently. Compounds such as thioglycolic acid (TGA), 3-mercaptopropionic acid (3-MPA), 2-mercaptoethanol, cysteamine, l-cysteine, or glutathione (GSH) ensure the stabilization of the nanocrystal in aqueous media through electrostatic charges, otherwise the quantum dots would aggregate and precipitate [32].

**Figure 4:**
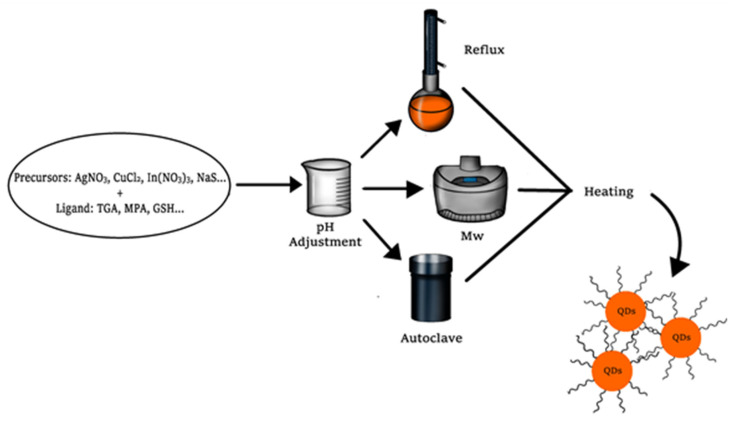
Synthesis of QDs by aqueous methods.

Quantum dot synthesis consists of two main processes, the formation of nuclei, also called nucleation, which is a fast step, and the growing of the nanocrystal, which is slow and ruled by an Ostwald ripening [33]. Each process occurs simultaneously in the synthesis and are promoted by temperature, which is applied in a reflux system, an autoclave, or using microwave radiation (Figure 4). In QDs, synthesis is important to control the variables involved in the synthetic protocols (concentration of precursors, concentration of ligand, time reaction, and temperature) because it affects the morphology, size of the nanoparticles, and the spectroscopic characteristics [34].

In a conventional reflux system, the metal salts are added to an aqueous media along with the capping agent; generally, the pH of the media is adjusted to basic values to promote the stability and enhance the quantum yield of the nanoparticle. Later, the chalcogenide is added, and the temperature is applied (generally around 100 °C). The reaction time is dependent on the characteristics required for the quantum dots, but generally does not require over 4 h. An inconvenience of reflux systems is that they do not permit an efficient control of QDs shape in their growth [35].

#### 2.1.1. Microwave Synthesis of QDs

Microwave-assisted synthesis of quantum dots is a technique where electromagnetic radiation in the wavelength range of 1 mm to 1 m is employed. The synthesis is based on the dielectric heating, which is the ability of a material (solvent) to convert microwaves into heat. This process occurs due to high-speed oscillations of dipolar molecules under the radiation [36]. In a typical microwave-assisted synthesis, metal precursors are mixed with the stabilizing agent in water, the pH of the solution is adjusted normally at basic values, and then the chalcogenide salt is added. Most of the microwave systems work at 0–1000 W power range and at 2450 MHz frequency and the common temperatures of synthesis are near to the water boiling point (90–110 °C). An advantage over reflux systems is that reaction times are considerably lower, allowing to obtain quantum dots at 5–30 min of irradiation [37].

Microwave-assisted synthesis allows to develop methods with high reproducibility to obtain quantum dots with narrow size distribution. This factor is important when QDs are incorporated in analytical sensing techniques [38].

#### 2.1.2. Solvothermal Synthesis

A third approach in which quantum dots can be synthesized in water is the solvothermal technique. In this method, an autoclave reactor is required to increase the pressure of the system and the boiling point of the solvent [39]. The term solvothermal makes refence to the reactions that take place at temperatures above the boiling point of the solvent; however, some authors use the term hydrothermal. As it was described in previous methodologies, the synthetic protocol is based on the mixture of metal precursors with the capping agent, the adjustment of pH, and the addition of the chalcogenide salt. The solution is transferred to a stainless-steel autoclave with an excellent sealing system that ensures the pressure in the medium. The normal conditions of synthesis are at 150 °C and 21 h of reaction according to Liu et al. [40].

#### 2.1.3. Transfer of QDs to Aqueous Media

Some synthesis strategies use organic solvents due to the high efficiencies in quantum yields obtained in the reactions (≈85%), but for chemical analysis purposes, in most cases, it is necessary to determine the analytes in aqueous matrices. Strategies of transference from organic solvents to water have been developed through the years and are based in hydrophilic molecules on the quantum dot’s surface [41].

The exchange of the surface ligand is a common strategy when tri-n-octylphosphine oxide (TOPO) is used as stabilizer of quantum dots. As the TOPO molecules are not covalently bonded to the QDs nanoparticles, molecules such as TGA can replace TOPO molecules on the surface of the nanoparticle. TGA contains a thiol group that is able to bind to the QDs and a carboxylic acid that can stabilize the nanoparticle in aqueous media. The method is considered a relatively simple process; nevertheless, it has several disadvantages, like the time employed in the two-step quantum dot obtention. Additionally, aggregation and oxidation can occur if the thiol group is not linked properly to the quantum dot surface, which leads to a reduction of the quantum yield [42].

#### 2.1.4. Synthesis of Core/Shell Quantum Dots

It is common to add a shell of a second semiconductor material to improve the spectroscopic properties of quantum dots; these structures are known as core/shell quantum dots [43]. Most ternary QDs use a ZnS, which has a larger band gap (3.7 eV [44] compared with AgInS_2_ 1.87 eV [45] and CuInS_2_ 1.23 eV [46]) to grow the shell to passivate the surface non-radiative recombination sites. This process prevents photo-blanching and passivates the surface traps, resulting in a %QY enhancement of QDs [47].

The synthesis of core/shell quantum dots is accomplished in two steps, and any of the previous hydrothermal methods can be easily used. After a complete formation of the core, the second metal cation (Zn, Cd, etc.) is added to the system and the time reaction is extended to ensure the formation of the shell around the core. Generally, when core/shell QDs are synthesized, the same chalcogenide is used in the core and in the shell, and therefore is added as excess reactant in the first step of the synthesis. In Table 1 are presented different conditions of synthesis of aqueous QDs.

As was mentioned before, to obtain core/shell quantum dots, a two-step process is required. For the aqueous strategies mentioned in this review, most of the methodologies described in Table 1 apply the same temperature in both steps, but the time required to obtain a stable shell regularly is longer than the time to obtain the core. This can be explained due the processes involved (nucleation: fast process and growth: slow process).

Particle size and maximum emission wavelength are related to the reaction time, showing bathochromic and hyperchromic effects. Nevertheless, microwave and solvothermal syntheses allow obtaining nanoparticles with higher %QY values within a couple of minutes, which is not observed in a reflux system [56]. Time is the critical factor in the synthesis because it can cause aggregation and oxidation of the precursors, leading to a decrease of the emission signal [37].

Most of the ternary QDs synthesized by reflux methods require higher reaction times to obtain nanoparticles below 4 nm. On the other hand, there are microwave methodologies that obtain nanoparticles of 8 nm at minor times. An important difference with respect to binary QDs is that the maximum wavelength emission is not completely related to the particle size. In this sense, the maximum emission wavelength may differ between particles and can be tuned in the visible range, although it is common to observe the emission at the near-infrared [37]. Morphology of ternary QDs is spherical or semi-spherical in most cases and the concentration of the stabilization agent plays an important role [57].

Among the different methodologies described, microwave synthesis seems to be the best option to obtain core/shell QDs with higher %QY (60% [38]), narrow size distribution, and that require less reaction time (minutes) compared to the reflux or solvothermal systems. Unfortunately, there are few methodologies that allow these %QY values, and it is important to develop more strategies to improve this parameter.

On the other hand, reflux synthesis allows to obtain QDs with an easier and cheaper equipment compared to the other two methodologies and even though the size distribution of the nanoparticles is broad, %QY is still significant for chemical analysis (10–50%). Some strategies are designed with QDs with relatively low %QY to promote an enhancement of the emission signal and register the increment of the signal, which cannot be significant in QDs with high %QY [58].

The use of solvothermal strategies (autoclave) for QDs synthesis has some advantages. The increment of the water boiling point at high pressure accelerates the QDs’ growth, and in consequence, the surface defects are reduced [59]. Siyu Liu et al. have synthesized CuInS_2_ QDs for 21 h to obtain the emission at near-infrared wavelengths, but it is possible to control the emission wavelength by reducing the synthesis time [60].

Ternary QD synthesis at a large-scale is still a challenge, due to the parameters obtained at laboratory conditions differing when they are produced in high amounts. The most common approach to large-scale synthesis is in pressure cookers, which is a variant of the solvothermal synthesis in which CuInS_2_, AgInS_2_, and CuInSe_2_ have been synthesized with a volume obtained of 4 L [61,62].

There are different interactions between the QDs and analytes; therefore, the synthetic protocol to be employed would depend on the analytical method design, and then it is necessary to evaluate the possible interactions and the changes produced on the spectroscopic signal [10].

## 3. Sensing Schemes

Due to their unique spectroscopic properties, ternary QDs have been widely used for chemical analysis of inorganic, organic, and biological molecules under different sensing schemes, depending on the binary interaction between the QDs’ surface and the analyte [11]. The objective of QDs as chemical sensors is to produce a change in their spectroscopic/fluorescence signal (at their maximum emission wavelength) because of a specific interaction with a given compound, to detect and correlate the changes to the analyte concentration [63].

The binary interaction between QDs and the analyte may result in a quenching or enhancing of the fluorescence signal of the nanoparticle due to recombination processes of the electron-hole pair. Analytical methodologies use these differences in the fluorescence intensity to quantify an analyte of interest. Therefore, it is important to understand the mechanism by which the process occurs to avoid possible interferences and make selective strategies. Quenching mechanisms such as photo-induced electron transfer (PET) and Förster resonance energy transfer (FRET) are more common in chemical analysis than enhancement of the signal due to passivation of the surface [64,65]. In the following, the mechanisms by which the fluorescence signal of QDs can be modified by an analyte are described

### 3.1. Photoinduced Electron Transfer

**Figure 5:**
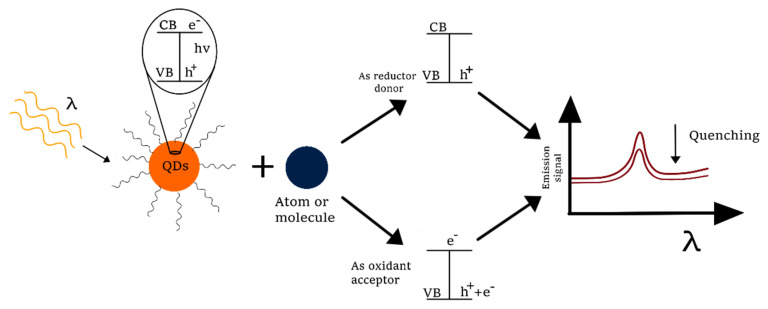
PET mechanisms of QDs as donor and acceptor (VB: valence band, CB: conduction band, e^−^: electron, h^+^: hole).

PET is a reversible quenching process where QDs absorb photons to promote an excited state. At this state, an electron occupies the highest energy molecular orbital (conduction band, HOMO) and a hole is formed in the valence band (LUMO), generating an electron-hole pair. In the PET process, the excited electron is transferred from the conduction band to an electron acceptor (A) as a reductant or accepts an electron in the hole from a donor (D) as an oxidant (Figure 5). In order to promote a PET process, it is necessary to have a difference in the redox potential in the donor and the acceptor energy levels [10].

The band gap in QDs is particle size-dependent; therefore, the electron transfer can be controlled by adjusting this parameter. Decreasing of a QD’s diameter results in an enhancement of the electron transfer [66]. Distance between the donor and acceptor also affects the rate of energy transfer. Different molecules can be determined in a direct way, such as metals and organic molecules with high redox potential. Another strategy is determination of analytes under an indirect system by recovering the fluorescence quenched [67].

Metal ions are common examples of analytes determined by ternary QD systems under PET principle. Castro et al. employed AgInS_2_ capped with 3-mercaptopropionic acid to determine Fe^2+^ in pharmaceutical formulations. Fe^2+^ quenched the emission signal of AgInS_2_ due to an electron transfer process obtaining a limit of detection of 0.6 mmol L^−1^ [68].

Liu et al. applied the reversibility of the quenching PET principle to determine biothiols (glutathione and l-cysteine) in human serum. The assay consisted of a quenching of CuInS_2_ QDs by Cu^2+^ ions due to PET process. The fluorescence was recovered after the addition of analytes due to the formation of a complex between the Cu^2+^ ion and the biothiols. The recovered signal is proportional to the analyte concentration [69].

Although it is common to observe PET process between QDs and a metal ion, it is possible to determine organic molecules with redox potentials under a PET scheme using ternary QDs. Shi et al. designed a methodology to determine dopamine employing AgInS_2_ capped with 3-mercaptopropionic acid. In the process, the AgInS_2_ QDs are excited with photons to promote an electron to the conduction band. The QDs, as the donor, transfers the electron to the dopamine-quinone molecule, inducing a quenching of the emission signal [70].

### 3.2. Förster Resonance Energy Transfer

**Figure 6:**
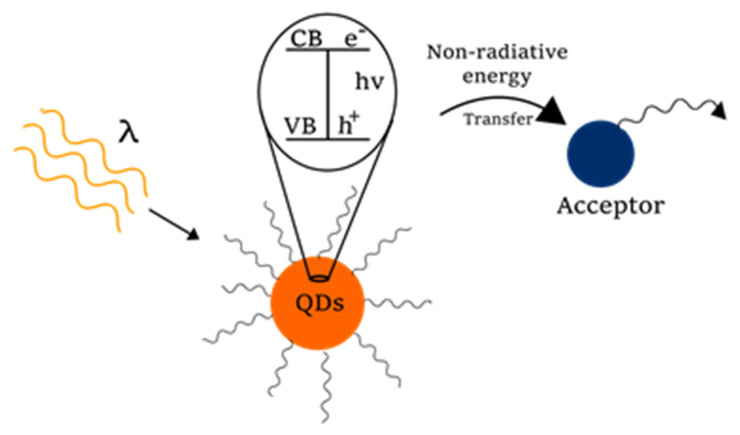
FRET mechanism of QDs and an acceptor (VB: valence band, CB: conduction band, e^−^: electron, h^+^: hole).

FRET is a non-radiative process where energy is transferred from a fluorescent donor in its excited state to an acceptor through dipole-dipole interactions (Figure 6). In order to promote the transference of energy, a proximity between donor and acceptor, generally under 10 nm of distance, is necessary, along with an overlap between the donor emission spectra and the acceptor absorption spectra [71].

QDs can be used as donors and acceptors in a FRET scheme but are preferred as donors due to their properties such as broad absorption spectra, narrow emission spectra, high extinction coefficients, high %QY, and the surface chemistry that allow the functionalization with different molecules. As was mentioned before, the emission of QDs is size-dependent and is tunable in the synthesis protocol; therefore, it is possible to obtain the desired emission spectra to promote the overlap required for FRET process. Along with the QD donors, it is necessary for an acceptor of the energy. In this sense, quenchers such as metal nanoparticles (AgNPs, AuNPs, etc.) or organic molecules can be used. Alternatives to quenchers are fluorescent molecules such as organic dyes and some proteins, which can act as acceptors in FRET systems [72,73].

The use of ternary QDs in sensing methodologies based on the FRET process has not been fully exploited and just a few articles describe it. Castro et al. developed a FRET system using AgInS_2_/ZnS QDs capped with D-penicillamide and gold nanoparticles (AuNPs) to determine atenolol. In the system, AgInS_2_/ZnS QDs are induced to an excited state to transfer energy to AuNPs, which results in an inhibition/quenching of the QDs’ fluorescence. When the analyte is added, it promotes the aggregation of AuNPs inhibiting the FRET process due to the lack of absorption of AuNPs and recovering of the fluorescence of AgInS_2_/ZnS QDs. Under this scheme, the authors reached a limit of detection of 1.05 mg L^−1^ and it was successfully applied for the determination of the analyte in pharmaceutical samples [74].

FRET systems between ternary QDs and fluorescent dyes have been also reported. Kuznetzova et al. developed a system between AgInS2/ZnS QDs (donor) and cyanine dyes (acceptor) Cy3 (3,3′-diethylthiacarbocyanine iodide) and Cy5 (3,3′-Diethylthiadicarbocyanine iodide) that generates a quenching of the fluorescence emission of QDs [75]. The principle of the FRET sensing scheme is an opportunity to develop further methodologies of analysis employing ternary QDs.

### 3.3. Other Mechanisms

Aggregation of the QDs is a non-reversible mechanism where the analyte interacts with the capping agent, inhibiting its stabilizing function and leading to a precipitation of QDs quenching the emission signal. Parani et al. developed a methodology for Cr(III) sensing employing AgInS_2_/ZnS QDs capped with glutathione. The interaction between QDs and Cr(III) resulted in a quenching due to the formation of a complex between the analyte and the glutathione on the surface of the QDs. The method reached a limit of detection of 0.51 nmol L^−1^ and showed a selectivity for the Cr(III) ion [50].

### 3.4. Enhancing Mechanisms

Surface chemistry of QDs is a significant factor to be considered when designing an analysis methodology. A core QD structure consists of (a) inner atoms that retain the geometry of the nanocrystal, (b) inorganic surface or outer atoms with a different morphology than the central atoms, and (c) ligand that stabilizes the nanoparticle. While the inner atoms establish the spectroscopical properties of QDs, the outer atoms can disturb them, due to the large number of atoms that are located on the surface [76].

During the synthesis protocols of QDs, it is usual to have defects on the surface such as dangling bonds, which create mid-gap states or traps that affect the electron-hole recombination and lead to a reduced photo-stability and %QYs [77].

**Figure 7:**
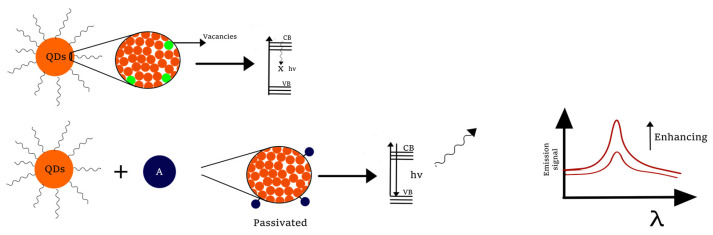
Resulting enhancing of QDs due to the passivation of traps (VB: valence band, CB: conduction band).

Although traps on the QDs’ surface affects considerably the photoluminescence of QDs, some strategies of analysis exploit this characteristic. Anions and cations on the surface are vacancies that can be bonded to specific molecules, eliminating the trap sites and passivating the QDs’ surface, resulting in an enhancement of the emission signal (Figure 7) [78].

Bambesiwe et al. developed a methodology in which employed AgInS_2_ QDs were capped with thioglycolic acid in order to determine ascorbic acid. After the addition of the analyte, the emission signal of QDs increased in the interval of 0.6–99 µmol L^−1^. This can be explained with the capacity of the ascorbic acid to form chelates with metals such as silver, which leads to the elimination of dangling bonds on the QDs surface, its passivation, and enhancement of the emission signal. The methodology reached a limit of detection of 26 nmol L^−1^ [55].

### 3.5. Functionalization of QDs

Ligands perform an important role in QD synthesis and define the physicochemical characteristics. Ligands selected and their concentration can influence the shape and size of the nanoparticle obtained. In QDs, ligands confer stability in aqueous media through electrostatic interactions; thiol-containing molecules with amine or carboxyl terminal groups (such as glutathione, 3-mercaptopropionic acid, mercaptoacetic acid, etc.) are commonly employed. The sulfide group is bonded to the metal ions on the surface of the QDs and the terminal group is then free to interact with the analyte [79].

Molecules such as peptides, proteins, aptamers, enzymes, antibodies, and nucleic acid can be coupled by covalent or non-covalent binding between the terminal group of QD ligands and a reactive group contained in the biomolecule: amino and carboxyl groups in peptides and proteins; phosphate, amine, and hydroxyl groups in DNA and aptamers. Functionalization process allows the promotion of a specific interaction between the QDs and an analyte, which results in a selective methodology of analysis [80,81].

Functionalization of ternary QDs is recent and there is a small number of articles that employs this principle for chemical sensing. Tianyu et al. synthesized CuInS_2_ functionalized with β-cyclodextrin and a selective aptamer by a covalent binding to detect adenosine-5′-triphosphate (ATP). The interaction between functionalized QDs and ATP results in an enhancement of the QDs’ fluorescence, obtaining a linear response from 6 to 1200 μmol L^−1^ and applied to determine ATP in human serum [82].

Ternary QDs can be functionalized with proteins. Liu et al. synthesized CuInS_2_ with bovine serum albumin by an amide formation between the carboxyl group from the 3-mercaptopropionic acid used as a QD ligand and an amine group from the protein. Functionalized QDs were employed to determine 2,4,6-trinitrophenol. The interaction between QDs and the analyte leads to a quenching of the fluorescence signal and was used to develop a methodology with LOD of 28 nmol L^−1^ [83].

AgInS_2_ have been used to develop immunoassays. Novikova et al. functionalized AgInS_2_/ZnS with immunoglobulin (IgG) selective to folic acid. Interaction of functionalized QDs and folic acid results in an inhibition of the fluorescence signal. The methodology was used to measure folic acid in fruit juice samples, achieving promising analytical parameters (LOD = 0.1 ng mL^−1^) [84].

An adequate selection of QDs may improve the analytical parameters of the sensitivity and LOD of the methodology designed. In general, methodologies based on QDs obtained lower LOD when using nanoparticles with higher %QY values. As an example, the use of CuInS_2_ QDs in the analysis of ascorbic acid reaches a limit of detection of 0.05 µM, while its analysis employing AgInS_2_ improves the LOD to 50 nM [55,85]. A similar response is observed in detection of Cu^2+^ in which the LOD obtained are 0.1 µM and 15 nM using CuInS_2_ and AgInS_2_, respectively [60,86].

In addition, QDs have been functionalized for biomedical applications. AgInS_2_ was functionalized with sgc8c aptamer to sense tumor cells [87], methotrexate for drug delivery [88], and DNA for potential biomedical applications [89]. Therefore, the functionalization of these novel nanoparticles is an opportunity area with several applications.

## 4. Application of Ternary QDs in Chemical Analysis

QDs have gained attention in developing analysis methods for many analytes in food, environmental, and pharmaceutical samples, although the binary core/shell of QDs such as CdTe/CdS and CdSe/CdS allows to obtain better emission signals. Ternary QDs are less toxic and permit the obtainment of adequate analytical parameters.

Sensing applications of ternary QDs are described in Table 2. It is shown that AgInS_2_ and CuInS_2_ are the predominant QDs employed for the development of analytical applications. When a core/shell structure is used, the main shell used is ZnS, leading to an increment in the detection limit reaching the order of nmol L^−1^ in most of the methodologies developed. Compared with Cd-containing QDs, core/shell ternary QDs are competitive: as an example, LOD obtained using AgInS_2_/ZnS in the determination of folic acid was (0.1 ng mL^−1^, 0.226 nmol L^−1^), while a strategy based on CdTe QDs obtained a LOD of 0.048 µmol L^−1^ [90]. Uric acid is another analyte which can be determined with CdTe QDs. In this methodology, the calibration curve was constructed from the emission signal quenched when the analyte is added, and the LOD reached was 0.1030 mol L^−1^, which is higher when CuInS_2_/ZnS is employed (LOD: 50 nmol L^−1^) [91].

Ternary QDs have been employed in food analysis to develop methodologies for the determination of contaminants (glufosinate) and nutrients (ascorbic acid and folic acid). The methodologies described commonly require pretreatment steps to remove possible interferents. Methodologies for determination of environmental pollutants have also been proposed using ternary QDs, mostly for toxic heavy metals (such as Co, Cr, Cd, Pb, Cu, etc.) and pesticides, in water matrices. Drug analytes are another category in which methodologies have been developed; in this case, the common matrices are pharmaceutical formulations and human serum.

Sensing mechanisms are diverse and depend mainly on the analyte determined. Most of them are based on the quenching of QDs, due to FRET, PET, and aggregation processes, although there are methodologies that take advantage of the reversibility of the PET mechanism to recover the emission signal. Enhancing and chemiluminescence are less used but are important mechanisms that can be exploited in further developments.

## 5. Conclusions and Trends

Ternary QDs are novel and promising tools for the development of chemical analysis methodologies. Their application in food, pharmaceutical, and environmental samples has grown during the last decade. Aqueous ternary QDs are competitive with binary Cd-containing QDs in terms of analytical parameters such as limit of detection, although in quantum yields and photoluminescence performance, there exist some deficiencies (surface traps, size optimization, and stability) that can be improved in the future.

Interaction mechanism principles between QDs and analytes are well described in the bibliography, but on occasion, it is difficult to identify the phenomena that is occurring; therefore, it is important to make efforts to point out and describe them clearly. AgInS_2_ and CuInS_2_ are the main QDs employed; there are still many faces to be exploited for ternary QDs in their photoluminescence performance, applications, and functionalization.

## Figures and Tables

**Table 1:** Hydrothermal synthesis conditions and spectroscopic parameters of ternary QDs.

Quantum Dots	Synthesis Strategy	Temperature-Time	Size (nm)	QY (%)	λ_em_ Max (nm)	Ref
CuInS_2_/ZnS	Reflux	95 °C 45 min (core) 80 min (Shell)	3.04 ± 0.47	12.3	708	[48]
CuInS_2_/ZnS	Reflux	100 °C 30 min core 30 min shell	1.8 ± 0.4	-	500–680	[49]
AgInS_2_/ZnS	Reflux	95 °C 45 min core 80 min shell	2.9	49.5	623	[50]
CuInS_2_	Solvothermal	150 °C 23 h	-	19.6	≈400	[51]
CuInSe_2_/ZnS	Reflux	100 °C 60 min for core 90 min for shell	4.19 (mean)	17.2	535	[52]
CuInS_2_/ZnS	Microwave	95 °C 10 min for core 5 min for shell	8.3	20.4	570	[53]
AgInS_2_	Hot injection method	90 °C 60 min	3–8	-	626	[54]
AgInS_2_	Reflux	95 °C 45 min	2.5	10.3	680	[55]
AgInS_2_/ZnS	Microwave	90 °C 30 min for core 100 °C 5 min for shell	2.7	60	625	[38]

**Table 2:** Analytical methodologies employing ternary QDs.

	Analyte	Ternary QDs	Sample	Interaction Mechanism	LOD	Ref
Food	Folic acid	AgInS_2_/ZnS	Fruit juices	Inhibition of fluorescence due to an antigen-antibody interaction (Immunoassay)	0.1 ng mL^−1^	[84]
	Glutathione	CuInS_2_	Tomatoes and urine	Recovery of the fluorescence quenched	73 nM	[92]
	Melatonin	AgInS_2_	Dietary supplements	Chemiluminescence	0.44 mg L^−1^	[93]
	Glufosinate	CuInS_2_	Tea leaves	Recovery of the fluorescence quenched	0.01 mg L^−1^	[51]
Environmental	Diniconazole	CuInS_2_	Tap water	Chemiluminescence	1 nM	[94]
	Zn^2+^	CuInS_2_	Tap water	Recovery of the fluorescence quenched	4.5 μM	[95]
	2,4,6-Trinitrophenol	CuInS_2_	Tap, spring, and waste water	Quenching of fluorescence	28 nM	[83]
	Cu^2+^ Cd^2+^	CuInS_2_	Tap and pond water	Cu^2+^: Quenching Cd^2+^: Enhancement	Cu^2+^: 0.037 mM Cd^2+^: 0.19 mM	[60]
Pharmaceutical	Ascorbic acid	CuInS_2_	Vitamin C tablets	Enhancement	0.05 mM	[85]
	Ciprofloxacin	AgInS_2_	Pharmaceutical tablets	Quenching	0.12 μg mL^−1^	[96]
	Sparfloxacin	CuInS_2_	Pharmaceutical tablets	Quenching	0.5 μg mL^−1^	[97]
	Atenolol	AgInS_2_/ZnS	Pharmaceutical formulations	FRET	1.05 mg L^−1^	[74]
Biological	Doxorubicin hydrochloride	CuInSe_2_/ZnS	Human serum	Quenching due to surface plasmon resonance effect	0.05 µM	[52]
	Adenosine-5′-triphosphate	CuInS_2_	Human serum	Enhancement	3 μM	[82]
	Heparin	CuInS_2_	Fetal bovine serum	Recovery of the fluorescence quenched	12.46 nM	[98]
	Histidine (His) Threonine (Thr)	CuInS_2_	Human serum	Recovery of the fluorescence quenched	His: 0.7 mM Thr: 2.0 mM	[69]
	Dopamine	CuInS_2_	Human serum	Quenching	0.2 µM	[99]
	Uric acid	CuInS_2_/ZnS	Human serum and urine	Enzymatic method, quenching	50 nM	[53]

## Data Availability

The data presented in this study are available on request from the corresponding author. The data are not publicly available due to privacy concerns.
